# The Generation of Tissue-Specific ECM Hydrogels From Melanoma and Associated Organs to Study Cancer Biology

**DOI:** 10.21769/BioProtoc.5586

**Published:** 2026-02-05

**Authors:** Yuval Mogilevsky, Chen Sharon-Yagol, Bar Manobla, Shahaf Saad, Ziv Raviv, Yuval Shaked

**Affiliations:** Rappaport Faculty of Medicine, Technion–Israel Institute of Technology, Haifa, Israel

**Keywords:** Extracellular matrix (ECM), Hydrogel, Melanoma, Tumor microenvironment, Decellularization, Collagen

## Abstract

The extracellular matrix (ECM) critically shapes melanoma progression and therapeutic response, yet commonly used matrices such as Matrigel fail to capture tissue- and disease-specific ECM properties. This protocol provides a streamlined and scalable method for generating murine, tissue-specific ECM hydrogels from skin, lung, and melanoma tumors, therefore overcoming the restricted materials of mouse-derived ECM. The workflow integrates tissue-tailored decellularization, lyophilization, mechanical fragmentation, pepsin digestion, and physiological polymerization to produce hydrogels that reliably preserve fibrillar collagen architecture and organ-specific ECM cues. Decellularization efficiency and ECM integrity are validated by DNA quantification, H&E staining, and Picrosirius Red staining analysis. These hydrogels provide a species- and tissue-matched platform for studying melanoma–ECM–immune interactions, pre-metastatic niche features, and therapy-induced ECM remodeling. Overall, this protocol offers a reproducible and physiologically relevant ECM model that expands experimental capabilities for melanoma biology and treatment-resistance research and that can be easily extended to other tumors and tissues.

Key features

• A miniaturized, tissue-specific workflow for generating ECM hydrogels from small murine skin, lung, and melanoma tissues, overcoming size limitations of existing protocols.

• Preservation of native ECM architecture using tailored decellularization steps validated by DNA quantification, H&E, and Picrosirius Red staining.

• A standardized digestion–gelation process optimized for heterogeneous and lipid-rich murine tissues, enabling reproducible hydrogel formation at defined ECM concentrations.

• A physiologically relevant platform capturing melanoma- and organ-specific ECM cues for studying ECM–tumor–immune interactions and therapy-induced remodeling.

## Graphical overview


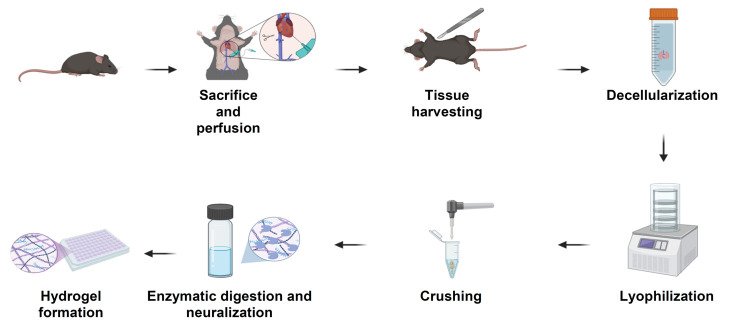


**Schematic overview of the extracellular matrix (ECM) hydrogel preparation protocol.** Following sacrifice and perfusion of mice, tissues of interest are harvested and subjected to a decellularization process to remove cellular material (24–48 h, depending on tissue type). Decellularized tissues are lyophilized for 24 h (lungs and tumors) or 48 h (skin), then mechanically crushed into a fine powder. The ECM powder is enzymatically digested under acidic conditions for 24 h (skin) or 48 h (lungs and tumors), neutralized, and adjusted to physiological ionic strength. The resulting pre-gel solution is incubated at 37 °C to form stable ECM hydrogels suitable for downstream biological applications.

## Background

Melanoma, the most lethal form of skin cancer, arises from transformed melanocytes and is characterized by high metastatic potential and marked immunogenicity [1]. Despite its substantial tumor mutational burden and responsiveness to immune checkpoint inhibitors, up to two-thirds of patients fail to benefit or eventually relapse following initial responses, highlighting the knowledge gaps in treatment resistance and disease progression [2].

Recent studies suggest that ECM remodeling in melanoma, among other cancers, plays a central role in invasion, metastasis, and immune regulation. Alterations such as increased matrix stiffness, collagen fiber alignment, and crosslinking, mediated by enzymes such as lysyl oxidases (LOX) and matrix metalloproteinases (MMPs), promote cancer cell dissemination and can physically hinder immune cell infiltration [3]. These changes not only facilitate melanoma progression but also contribute to phenotype switching and resistance to targeted or immune-based therapies. Preclinical studies targeting ECM components, such as integrins, hyaluronan, and MMPs, have demonstrated potential synergy with immunotherapy, pointing to the ECM as a promising therapeutic co-target in melanoma [4]. Therefore, the advancement of new tools to study ECM in melanoma can advance melanoma research and serves as a basis for the development of new treatments for melanoma.

The composition and organization of ECM proteins vary across tissues, reflecting their unique properties and functional requirements [5]. Additionally, the ECM undergoes dynamic changes over time, adapting to tissue needs and the embedded cells within each organ [6]. In cancer, the ECM plays a pivotal role, usually supporting tumor development and progression. Unlike normal tissues, the ECM of solid tumors exhibits significant alterations in protein composition and structure [7]. Changes in the ECM occur not only in the primary tumor but also in distant organs [8]. ECM remodeling in distant organs creates a supportive environment for disseminated cancer cells, creating a pre-metastatic microenvironment, allowing cancer cells to home to such sites and colonize there. These ECM alterations are driven by factors secreted by the primary tumor, such as extracellular vesicles and LOX, and involve interactions among resident stromal cells and recruited immune cells [9].

Current in vitro ECM models, including collagen gels, Matrigel, and synthetic hydrogels, do not reflect the diverse ECM compositions of melanoma across anatomical or metastatic sites [10,11]. While tissue-derived hydrogels generated from human or porcine organs provide more physiological ECM environments [12], they are limited by availability, batch variability, and lack of compatibility with murine experimental systems. Mouse-derived ECM hydrogels would enable species-matched studies, facilitate integration with in vivo perturbations, and support mechanistic work in genetically engineered or syngeneic models. However, their use has been restricted by the small size of murine tissues, which often yields insufficient material for conventional decellularization and hydrogel preparation [13].

This protocol addresses these limitations by establishing a miniaturized, tissue-specific workflow for generating ECM hydrogels from a small quantity of murine skin, lung, and melanoma tumors. The method preserves native ECM cues and supports downstream applications for modeling melanoma–ECM–immune interactions, metastatic niche conditions, and therapy-induced ECM remodeling [14,15]. Beyond melanoma, the same workflow can be adapted to other solid tumor types; we have successfully applied similar decellularization steps to LLC, 4T1, and PyMT tumors, indicating broad applicability across murine cancer models.

## Materials and reagents

**Biological materials**

1. YUMM 1.7 murine melanoma cells (ATCC, CRL-3362)

2. B16-F10 murine melanoma cells (ATCC, CRL-6475)

3. C57BL/6 mice (Harlan Laboratories Israel)

**Reagents**

1. Double-distilled water (DDW)

2. 1× phosphate-buffered saline (PBS) (Sigma-Aldrich, catalog number: L0615-500ML)

3. 10× PBS (Sigma-Aldrich, catalog number: D1408-500ML)

4. Triton X-100 (Sigma-Aldrich, catalog number: T8787)

5. Ammonium hydroxide solution 25% (Biolab, catalog number: 125050100)

6. DNase I powder 3,520 U/mg (Worthington, catalog number: LS002139)

7. Ethanol, absolute >99.8% (Gadot Group, catalog number: 64-17-5)

8. Hydrogen peroxide (H_2_O_2_), 33% (Panreac, catalog number: 131077.1211)

9. Acetic acid glacial 99.7% (BioLab, catalog number: 107052100)

10. Tris base (Sigma-Aldrich, catalog number: T1503)

11. Ethylenediaminetetraacetic acid (EDTA) (Sigma-Aldrich, catalog number: EDS-100G)

12. Trypsin 0.25%–EDTA (0.038%) in HBSS (IMBH, catalog number: L0931-500ML)

13. Pepsin from porcine gastric mucosa powder (Sigma-Aldrich, catalog number: P7012-1G)

14. Sodium hydroxide pearls (NaOH) (Biolab, catalog number: 1908059100)

15. Hydrochloric acid 32% (HCl) (BioLab, catalog number: 846050100)

**Solutions**

1. Triton X-100/ammonium hydroxide solution (see Recipes)

2. Triton X-100/0.26% EDTA/0.69% Tris (see Recipes)

3. 0.4% peracetic acid/4% ethanol solution (see Recipes)

4. Pepsin solution (see Recipes)

5. DNase I solution 50 U/mL (see Recipes)

6. 70% ethanol (see Recipes)

7. 3% H_2_O_2_ (see Recipes)

**Recipes**

**1. Triton X-100/ammonium hydroxide solution**

**Table:** 

Reagent	Final concentration	Quantity or volume
Triton X-100	1%	10 mL
25% ammonium hydroxide	0.004%	4 mL
DDW	n/a	986 mL

**2. Triton X-100/0.26% EDTA/0.69% Tris**

**Table:** 

Reagent	Final concentration	Quantity or volume
Triton X-100	1%	10 mL
EDTA powder	0.26%	2.6 g
Tris base	0.69%	6.9 g
DDW	n/a	990 mL

**3. 0.4% peracetic acid/4% ethanol solution**

**Table:** 

Reagent	Final concentration	Quantity or volume
Acetic acid glacial	0.4%	1 mL
33% H_2_O_2_	0.03%	1 mL
Ethanol, absolute	4%	40 mL
DDW	n/a	958 mL

**4. Pepsin solution**

**Table:** 

Reagent	Final concentration	Quantity or volume
Pepsin from porcine gastric mucosa	1 mg/mL	5 mg
0.01 M HCl	0.01 M	5 mL

*Note: This recipe is calculated based on preparing solutions for processing 50 mg of dry ECM powder. Volumes and reagent quantities should be scaled accordingly if processing larger or smaller tissue amounts. Ensure precise weighing of the ECM powder prior to preparation and adjust all components proportionally to maintain the specified final concentrations.*

**5. DNase I solution 50 U/mL**

**Table:** 

Reagent	Final concentration	Quantity or volume
DNase I powder 3,520 U/mg	50 U/mL	0.071 mg
1× PBS	n/a	5 mL

**6. 70% ethanol**

**Table:** 

Reagent	Final concentration	Quantity or volume
Ethanol, absolute >99.8%	70%	350 mL
DDW	n/a	150 mL

**7. 3% H_2_O_2_**

**Table:** 

Reagent	Final concentration	Quantity or volume
33% H_2_O_2_	3%	45.5 mL
DDW	n/a	454.5 mL

*Note: All recipes should be made fresh.*

**Laboratory supplies**

1. Eppendorf tubes 1.7 mL (Corning Inc., catalog number: 28324032)

2. Scintillation vials (Sigma-Aldrich, catalog number: 986546)

3. Depilatory cream

4. 27G needle (Bactlab, catalog number: BD300635)

5. Scissors (Sigma-Aldrich, catalog number: 41122408)

6. Butterfly needle (Bar Naor, catalog number: BNSCLPV23g)

7. 20 mL syringe (BD Emerald, catalog number: 307736)

8. 200 μL pipette tips (TH Geyer, catalog number: 7695884)

9. 50 mL conical tubes (Greiner Bio-One, catalog number: 227261)

## Equipment

1. Lyophilizer (Labogene, model: CoolSafe 110-4, catalog number: 7001000115)

2. Shaker-incubator (Benchmark, catalog number: H2010)

3. Horizontal shaker (ELMI, catalog number: S-3.02)

4. Cellular centrifuge (Heraeus, catalog number: PICO17)

5. Intelli-Mixer (ELMI, catalog number: RM-2L)

6. Handheld tissue homogenizer (VISOSCI, catalog number: B0CG4KPQW5)

7. 37 °C dry incubator (Heracell, catalog number: 150i)

8. -80 °C freezer

9. Refrigerator (2–8 °C)

## Procedure

*Note: The following protocol is divided into two parts. Part I is the decellularization process for tumor, lung, and skin tissues. Part II is the freeze-drying and gelation processes. The overall protocol is illustrated in the graphical abstract. It is important to note that the procedure does not require aseptic conditions or prior filtration of solutions and reagents, as the final pre-gel solution can be irradiated with UV light for 15 min before use in downstream assays.*

**Part I. Decellularization**

Decellularization is the essential first step in producing intact, tissue-specific ECM for hydrogel preparation. Its goal is to efficiently remove cellular content without disrupting the native structural and biochemical composition of the matrix. Because tissues differ significantly in cellularity, density, and ECM architecture, we employ modified protocols for tumors, lungs, and skin to ensure consistent removal of cells while preserving ECM integrity for downstream freeze-drying and gelation.

**A. Decellularization of tumors and lungs**

1. Euthanize mice according to institutional animal care and use committee (IACUC) guidelines.

2. Cardiac perfusion: Insert a butterfly needle into the left ventricle of the mouse heart and make a small cut on the right ventricle.

3. Connect a 20 mL syringe to the tubing distal edge and flow DDW through it using low-to-medium pressure.

4. Remove the lungs or tumor and soak in 50 mL of DDW within a 50 mL tube. Rotate for 2 h at 4 °C using an Intelli-Mixer device at 30 rpm.

*Note: Tumors were generated by injecting YUMM 1.7 or B16-F10 melanoma cells intradermally into the dorsal flank of C57BL/6 mice (typically, 5 × 10^5^ cells in 100 μL of HBSS per mouse). Tumors were allowed to grow until they reached approximately 1,500 mm^3^.*

5. After 2 h, replace the DDW.

6. Rotate further for at least 48 h at 4 °C and 30 rpm. Replace the DDW once every 24 h.

*Note: Proceed to the next step only when the lungs or tumors no longer appear reddish.*

7. Exchange the DDW with 50 mL of the Triton X-100/ammonium hydroxide solution. Rotate on Intelli-mixer for an additional 48 h at 4 °C and 30 rpm.

*Note: Triton X-100 solubilizes cellular membranes, while ammonium hydroxide helps remove nuclear material and loosens tissue architecture.*

8. Monitor the clearness of the lungs or tumors. If the organs are not clear yet, replace the Triton X-100/ammonium hydroxide solution with a fresh solution and continue to rotate for an additional 24 h.

9. Wash with DDW for 15 min × 3 times on a horizontal shaker at room temperature (RT) and 300 rpm.

10. Fat removal (for tumor only, performed at RT): Change to 50 mL of 70% ethanol and rotate for an additional 12 h, followed by 15 min in 50 mL of 3% H_2_O_2_, and an additional 2 h in 50 mL of 0.4% peracetic acid solution/4% ethanol. Follow with three washes with DDW for 15 min, all performed on a horizontal shaker at RT and 300 rpm.

*Note: 70% ethanol removes lipids and helps dehydrate tissues, H_2_O_2_ oxidizes and bleaches residual pigments, and peracetic acid acts as a potent disinfectant and removes residual organic material while preserving ECM structure.*

11. Insert tissues in 5 mL of DNase I solution and rotate at 150 rpm on a shaker-incubator at 37 °C for 1 h.

*Note: DNase I digests residual nucleic acids, ensuring complete removal of DNA before downstream processing.*

12. Wash with DDW for 2 h on an Intelli-mixer device at 4 °C and 30 rpm.

13. After 2 h, replace the DDW with fresh DDW and wash for another 24 h at 4 °C and 30 rpm.

14. Transfer decellularized tissue sections into a 1.7 mL Eppendorf and add 200 μL of PBS 1×.

15. Using scissors, cut the sections into small pieces.

16. Centrifuge at 11,000× *g* for 2 min, then gently discard as much fluid as possible.

17. Store at -80 °C.

*Note: Issues can be stored for up to 1 year at this point.*

**B. Decellularization of skin**

1. Euthanize according to institutional animal care and use committee (IACUC) guidelines.

2. Insert a butterfly needle into the left ventricle of the mouse heart and make a small cut on the right ventricle.

3. Connect a 20 mL syringe to the tubing distal edge and flow DDW through it at low-medium pressure.

*Note: Relevance of perfusion: Although the skin is collected externally, cardiac perfusion is performed to remove blood from dermal and subdermal vessels. This reduces residual hemoglobin and cellular components, improves decellularization efficiency, and enhances the clarity and consistency of the resulting ECM for hydrogel preparation.*

4. Gently shave the desired skin section and apply depilatory cream for 1–2 min.

5. Collect the skin section by separating it gently from the underlying tissues.

6. Separate the subcutaneous layer manually using a scalpel.

7. Cut the skin into 1 cm^2^ rectangles, put in a 1.7 mL Eppendorf tube, and store at -80 °C until use.

*Note: Tissues can be stored for up to 1 year at this point.*

8. Place 1–2 skin sections into one 50 mL tube and incubate sequentially in 50 mL of each of the following solutions on a horizontal shaker (300 rpm) at RT (unless otherwise specified):

a. 0.25% Trypsin-EDTA (0.038%) in HBSS: 6 h at 37 °C and 300 rpm.

b. DDW: 15 min × 3 washes.

c. 70% Ethanol: 10–12 h (once).

d. 3% H_2_O_2_: 15 min (once).

e. DDW: 15 min × 2 washes.

f. 1% Triton X-100/0.26% EDTA/0.69% Tris: 6 h (once) followed by overnight incubation.

g. DDW: 15 min × 3 washes.

h. 0.4% peracetic acid/4% ethanol: 2 h (once).

i. PBS: 15 min × 2 washes.

j. DDW: 15 min (once).

k. DNase I solution (50 U/mL): 1 h at 37 °C and 300 rpm.

l. PBS: 15 min × 2 washes.

m. DDW: 15 min × 3 washes, followed by overnight incubation.

9. Transfer the decellularized tissue sections into a 1.7 mL Eppendorf tube and add 200 μL of PBS.

10. Using scissors, cut the section into small pieces.

11. Centrifuge at 11,000× *g* for 2 min, then gently discard the fluid as much as possible.

12. Store at -80 °C.

*Note: Tissues can be stored for up to 1 year at this point.*

**Figure 1.**
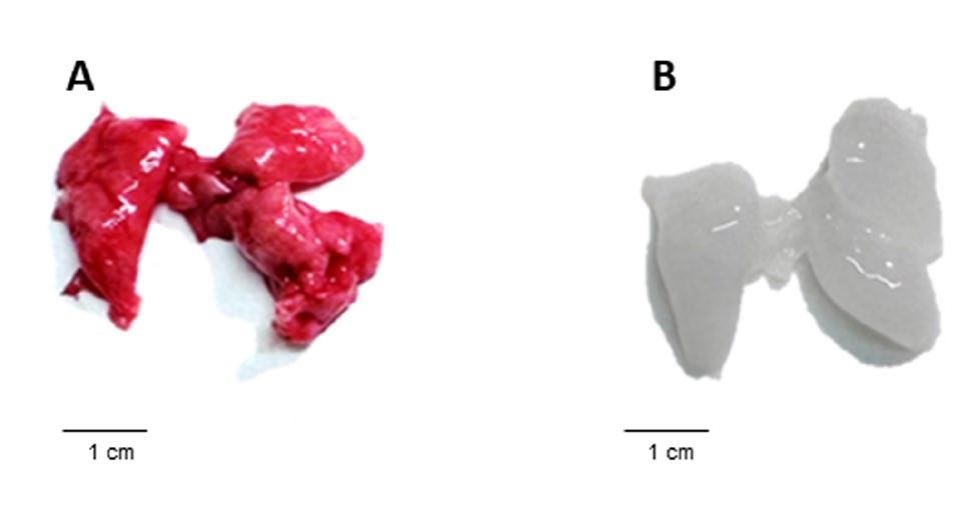
Gross morphology of murine lungs before and after decellularization. (A) Mouse lung tissue prior to decellularization, showing a characteristic red coloration of intact cellular and vascular components. (B) Lungs following completion of the decellularization protocol, demonstrating loss of cellular material and blood components, resulting in a pale, translucent tissue. This comparison visually illustrates the effectiveness of the decellularization process described in the protocol.

**Figure 2.**
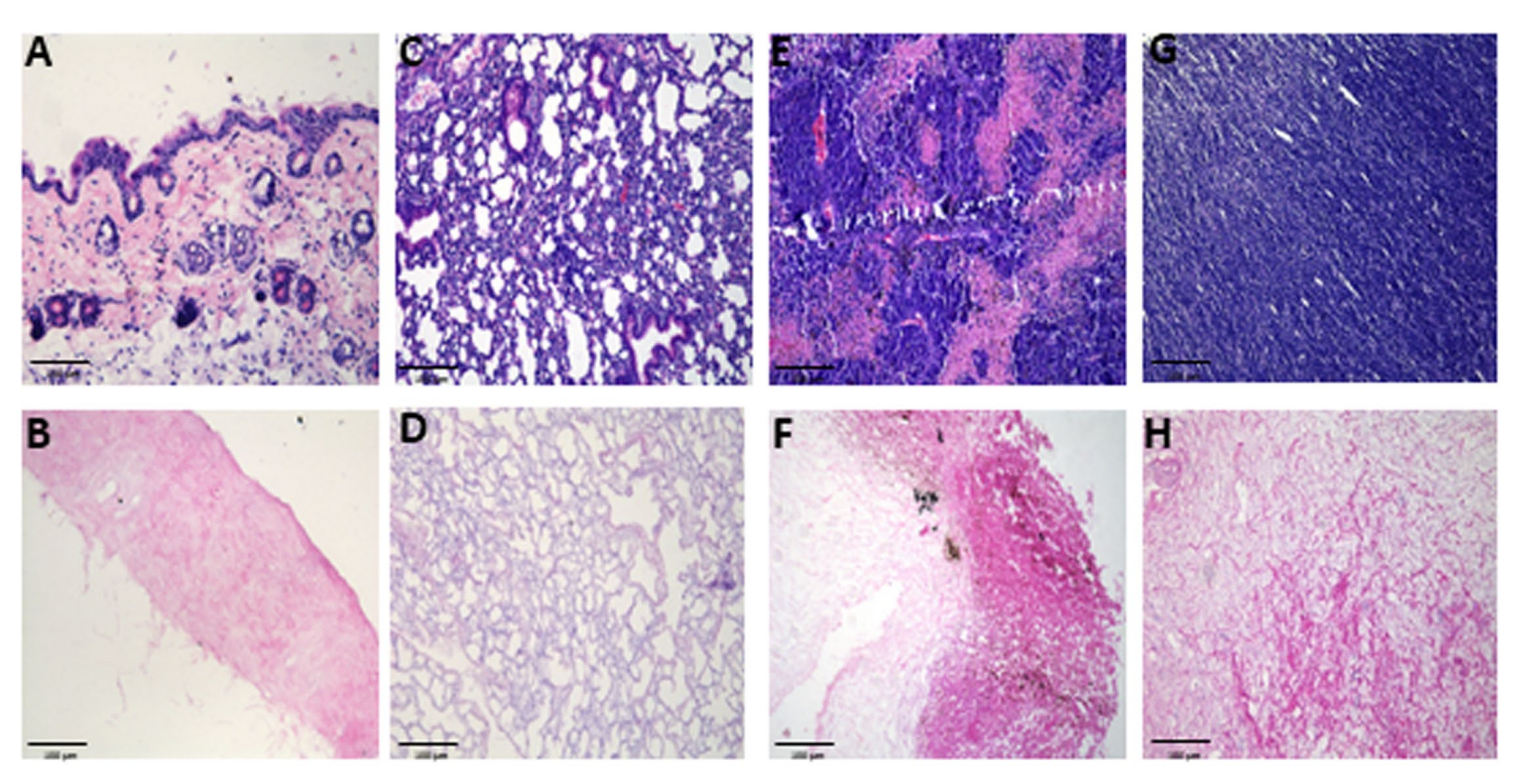
Histological evaluation of native and decellularized tissues. Representative hematoxylin and eosin (H&E) staining images of murine tissues: skin (A–B), lung (C–D), B16-F10 (E–F), and YUMM 1.7 (G–H) melanoma tumors, before (top row) and after decellularization (bottom row). n ≥ 3 samples/group, imaged at 20× magnification, scale bar, 100 μm. Decellularized matrices retained overall extracellular structure while showing effective removal of cellular material. In B16-F10 tumors (F), residual pigment is evident, representing melanin spillover from melanoma cells, which persists despite decellularization.

**Figure 3.**
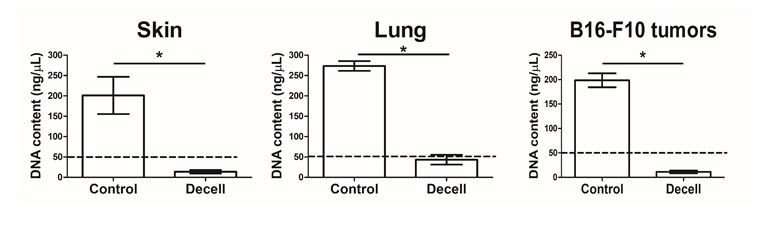
DNA content of native vs. decellularized tissues. DNA concentration (ng/μL) of murine skin (A), lung (B), and B16-F10 tumors (C) before (Control) and after (Decell) the decellularization process (n = 3 samples/group, mean ± SD). Genomic DNA was isolated using the NucleoSpin Tissue kit (Macherey-Nagel) according to the manufacturer’s protocol and quantified using NanoDrop. All tissues show a significant reduction in DNA following the decellularization process, confirming effective removal of cellular material. The dashed line represents the threshold of the effective decellularization process, as previously published [16,17]. *p < 0.05, using an unpaired two-tailed t-test.

*Note: An example of a successful decellularization process in lungs is provided in Figure 1, where the lungs no longer appear reddish. In addition, the decellularization process can be evaluated using hematoxylin and eosin (H&E) staining to determine the lack of nuclei and cells in the decellularized tissue (Figure 2). Moreover, additional validation for the decellularization process can be done by analyzing the content of DNA in the decellularized tissue (Figure 3).*

**Part II. Freeze-drying and gelation**

The purpose of this step is to process the decellularized tissue into a soluble form that can subsequently self-assemble into hydrogels. The freeze-dry procedure removes water content while preserving ECM proteins, ensuring long-term storage and accurate downstream quantification. Mechanical fragmentation and pepsin digestion are then used to solubilize the ECM into small protein fragments. After pH neutralization and adjustment to the desired concentration, the solution is incubated at 37 °C, enabling ECM proteins to reassemble and form hydrogels.

In practice, decellularized tissues are rapidly transferred from the freezer to the lyophilizer to prevent thawing. After lyophilization, samples are mechanically crushed (e.g., with a handheld tissue grinder) and digested in pepsin solution. The digested ECM is neutralized with NaOH and diluted to ~8 mg/mL protein. For fat-rich samples, especially dermal/subdermal tumors, a further clarification step is included.

1. Punch Eppendorf tube cups with a 27G needle (7–10 holes). Cut out the punched cup and place it on the tissue-containing Eppendorf tubes.

2. Quickly apply the samples to a lyophilizer at -110 °C for 24 h (tumors and lungs) or 48 h (skin). When the lyophilization ends, quickly remove the punched cups and close the Eppendorf tube with the original cups.

3. Put the tubes at -80 °C.

*Note: The samples can be kept in a -80 °C freezer for several months.*

4. Before crushing, weigh the tissues and record the weight.

5. Grind the tissue using a handheld tissue grinder by adding 200 μL of pepsin solution to the Eppendorf tube containing the tissue sample. Grind until the solution is homogeneous. If needed, add more pepsin solution and grind until it becomes homogeneous. Mark how much pepsin solution is being used during the grinding process.

6. Transfer the ground tissue into a scintillation vial.

**Critical:** After the tissue is transferred to the scintillation vial, add pepsin solution to achieve a final ratio of 10 mg of lyophilized decellularized tissue powder per 1 mL of pepsin solution (if this ratio has already been achieved during the grinding process, no additional pepsin is required).

7. Incubate for 24 h (skin) or 48 h (lungs and tumors) on a horizontal shaker at 150 rpm at RT.

**Critical:** After 24 h, titrate the pepsin-digested solution to pH 7.2–7.4 using 0.1 M NaOH. Add NaOH gradually in small increments to avoid rapid pH shifts and carefully monitor the pH to prevent exceeding pH 7.5. Record the volume of NaOH added.

8. Add sterile 10× PBS equivalent to one-ninth of the final solution volume.

9. Add sterile 1× PBS to reach the target ECM concentration (typically ~8–10 mg/mL protein).

10. Optional clarification step: If visible debris is present, transfer the digested solution to a new 1.7 mL Eppendorf tube and centrifuge at 11,000× *g* for 2 min. Carefully collect the supernatant to enhance gel clarity.

11. The pre-gel can be stored at 4 °C for up to 1 week before use.

12. Transfer the pre-gel solution to a required tissue culture plate, transwell insert, or other platform according to the intended downstream application. Incubate at 37 °C for 1 h to allow self-assembly and hydrogel formation.

a. Recommended hydrogel volumes and thicknesses:

i. 96-well plates: 30–60 μL per well → ~0.5–1 mm gel thickness.

ii. 24-well plates: 100–200 μL per well → ~1.5–2 mm thickness.

iii. Transwell inserts: 50–100 μL per insert.

iv. In vivo injections (mice): 100–300 μL per injection site.

*Notes:*

*1. Gelation behavior: Gels ≥ 8 mg/mL are formed consistently and readily at 37 °C. Concentrations below ~3–4 mg/mL may result in incomplete polymerization.*

**Figure 4.**
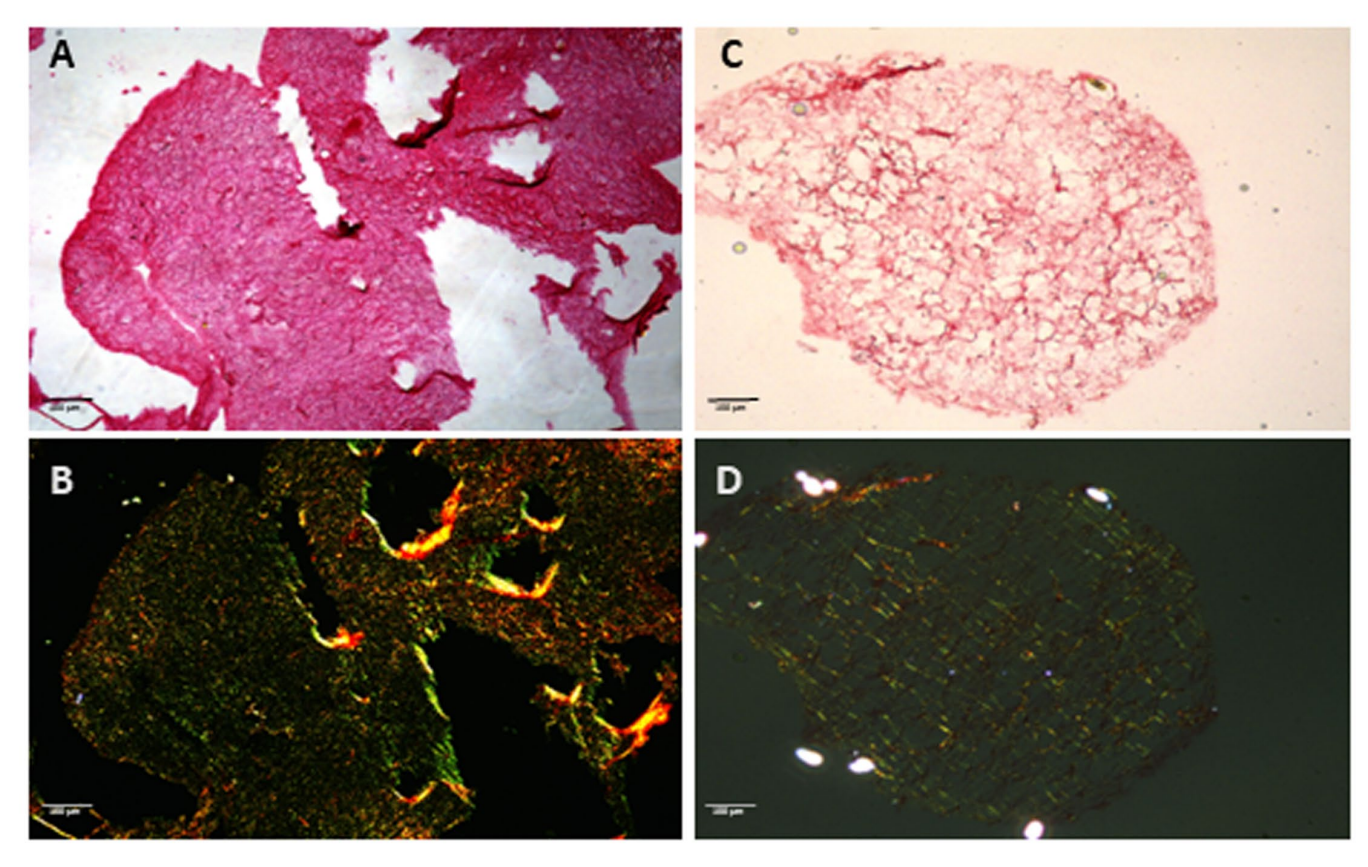
Collagen assessment in extracellular matrix (ECM) hydrogels by Sirius Red staining. Sirius Red staining of hydrogels derived from skin (A–B) and lung (C–D) decellularized murine tissues. Images were taken at brightfield (top row) and corresponding polarized light highlighting collagen birefringence (bottom row). Collagen fibers are preserved in both preparations, confirming retention of structural ECM components after decellularization and hydrogel formation. Under polarized light, skin-derived hydrogels display thicker, more organized collagen bundles, whereas lung-derived hydrogels exhibit finer, loosely arranged fibers, reflecting site-specific ECM architecture. Scale bar, 100 μm.

*2. The hydrogel end-product can be stained or used for various applications, such as Sirius Red staining (Figure 4).*

## Validation of protocol

The reproducibility and robustness of this protocol were validated through independent preparations of ECM hydrogels derived from murine skin, lung, and melanoma tumors. Each tissue type was processed in at least three independent biological replicates, following identical decellularization, lyophilization, digestion, and polymerization steps. The following validation steps were carried out: (A) An effective decellularization process was confirmed by histological evaluation (H&E staining) showing complete removal of nuclei and cellular material. Representative microscopy images of the different tissues were obtained from three replicates. (B) DNA quantification in decellularized tissue demonstrated a significant reduction in DNA content after the decellularization process. The experiment was carried out in triplicate (Figure 3). (C) Collagen preservation and fibrillar organization after hydrogel formation were verified using Picrosirius Red staining under polarized light, revealing site-specific ECM architectures that remained consistent between preparations. Representative images from three to four replicates are shown (Figure 4).

## General notes and troubleshooting

**General notes**

**Figure 5.**
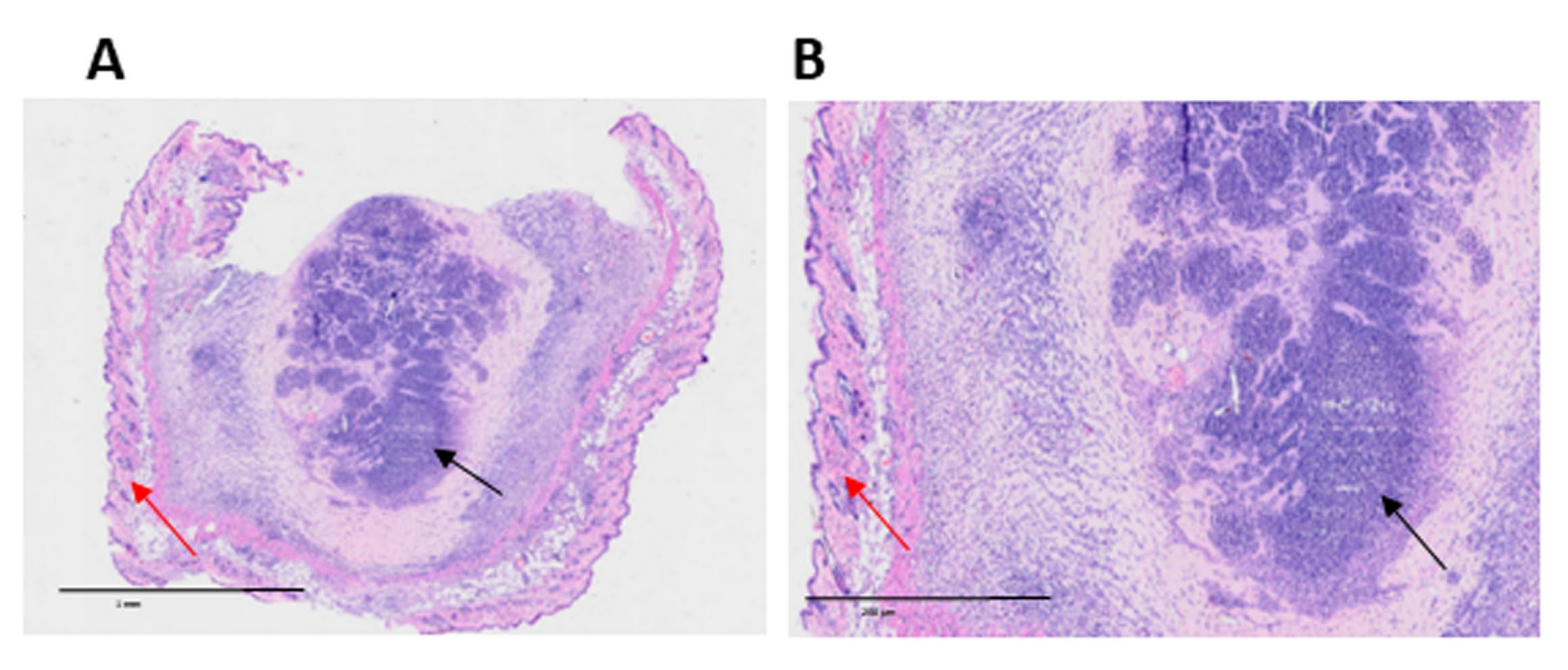
Host cell infiltration into intradermally injected extracellular matrix (ECM) hydrogel. Representative hematoxylin and eosin (H&E) staining images of murine skin, 7 days after intradermal injection of 300 μL of B16-F10 tumor-derived hydrogel. The central region corresponds to the hydrogel (black arrows), surrounded by normal skin tissue (red arrows). Infiltrating host cells are visible within the hydrogel, indicating cellular migration into the implanted matrix. (A) 10× magnification; scale bar, 1 mm. (B) Zoom magnification based on OlyVIA software; scale bar, 200 μm.

1. Tissue variability and protocol limitations: The biochemical composition and stiffness of ECM differ between tissues and even between animals, affecting digestion efficiency, gelation, and downstream cellular behavior. Therefore, results obtained using ECM from a specific site or species may not be directly comparable across models. This protocol is optimized for murine tissues and may require adjustments (e.g., detergent strength or digestion time) for other organs or species.

2. Compatibility with downstream assays: The resulting hydrogel is compatible with spheroid embedding, migration assays, immunostaining, and protein extraction.

3. Compatibility with in vivo and histological workflows: The hydrogels can be injected intradermally or subcutaneously, forming stable, retrievable depots that remain identifiable and compatible with standard histopathological staining (Figure 5).

4. Applicability to additional tumor models: This protocol is compatible with a wide range of murine solid tumors. We successfully applied the decellularization and hydrogel-generation workflow to LLC (Lewis Lung Carcinoma), 4T1 mammary carcinoma, and PyMT mammary tumors, achieving effective decellularization with only minor adjustments in detergent exposure time based on tumor cellularity and lipid content. This indicates the flexibility and robustness of the protocol for use beyond melanoma-derived tissues.

**Troubleshooting**

**Problem 1:** Incomplete decellularization.

Possible causes: Insufficient duration of detergent incubation, inadequate perfusion leading to retained blood, tissue pieces cut too large, limited detergent penetration, low agitation speed, or insufficient solution volume.

Solutions: Extend immersion in Triton X-100/ammonium hydroxide solution by 24–48 h and replace with fresh solution, ensure proper perfusion before tissue collection, and cut tissues into small pieces (2–3 mm).

**Problem 2**: Inhomogeneous hydrogel formation.

Possible causes: Incomplete solubilization of the ECM or poor mixing procedure during neutralization.

Solution: Centrifuge at 11,000× *g* for 2 min.

**Problem 3:** Batch-to-batch variation in hydrogel stiffness.

Possible causes: Variable tissue composition, incomplete lyophilization, or inconsistent ECM dry weight normalization.

Solutions: Standardize tissue weight-to-volume ratios and record all intermediate weights; normalize digestion to 10 mg of ECM powder per milliliter of pepsin solution.

**Problem 4:** Hydrogel does not polymerize at 37 °C.

Possible cause: Over-digestion or low ECM concentration (<3 mg/mL).

Solution: Reduce digestion time or prepare a more concentrated ECM solution. Verify if the pH is within the range of 7.2–7.6.
